# Negative Emotional Arousal Impairs Associative Memory Performance for Emotionally Neutral Content in Healthy Participants

**DOI:** 10.1371/journal.pone.0132405

**Published:** 2015-07-17

**Authors:** Jonathan Guez, Rotem Saar-Ashkenazy, Liran Mualem, Matan Efrati, Eldad Keha

**Affiliations:** 1 Beer-Sheva Mental Health Center, Beer-Sheva, Israel; 2 Department of Psychology, Achva Academic College, Beer-Tuvia, Israel; 3 Department of Cognitive Neuroscience and Zlotowski Center for Neuroscience, Ben-Gurion University of the Negev, Beer-Sheva, Israel; 4 Department of Psychology and the School of Social-work, Ashkelon Academic College, Ashkelon, Israel; 5 Department of Psychology, Tel-Aviv University, Tel-Aviv, Israel; University of L'Aquila, ITALY

## Abstract

The effect of emotional arousal on memory presents a complex pattern with previous studies reporting conflicting results of both improved and reduced memory performance following arousal manipulations. In this study we further tested the effect of negative emotional arousal (NEA) on individual-item recognition and associative recognition of neutral stimuli in healthy participants, and hypothesized that NEA will particularly impair associative memory performance. The current study consists of two experiments; in both, participants studied a list of word-pairs and were then tested for items (items recognition test), and for associations (associative recognition test). In the first experiment, the arousal manipulation was induced by flashing emotionally-negative or neutral pictures between study-pairs while in the second experiment arousal was induced by presenting emotionally-negative or neutral pictures between lists. The results of the two experiments converged and supported an associative memory deficit observed under NEA conditions. We suggest that NEA is associated with an altered ability to bind one stimulus to another as a result of impaired recollection, resulting in poorer associative memory performance. The current study findings may contribute to the understanding of the mechanism underlying memory impairments reported in disorders associated with traumatic stress.

## Introduction

Understanding the mechanism underlying the effect of stress on memory carries practical and theoretical considerations, with research in this field have made an important progress by the discovery that glucocorticoids (GCs), stress hormones released from the adrenal cortex, bind to specific receptors in the hippocampus [1], a key region for declarative/episodic memory [2], [3]. Notwithstanding, studying the specific contribution of emotional arousal, one of the key features of traumatic stress, carries important implications for understanding normal human memory as well as pathological reactions to stress. While most studies use different stress manipulations (cortisol administration or psychological stress manipulations) to test the effect of stress on memory, only a limited number of existing studies of this concept tested the effect of negative emotional arousal (NEA, i.e. a state of being mentally and physically reactive to negative arousing pictures, as defined by independent participants’ subjective ratings of valence and arousal, and has been cross validated, see [4].

According to the dual-process theory, episodic memory is based on two independent contributing processes, familiarity (which is associated with a vague experience of remembering and is relatively automatic in nature) and recollection (which involves executive functioning, and is associated with a clear sense of remembering, see [5], [6]. During familiarity the retrieved information is based on the intensity of the impression that the unit has been previously perceived; while in the case of recollection, an encoded unit is retrieved together with its contextual information (e.g. time and/or place). This distinction, which is supported by numerous cognitive and neuroimaging studies, postulates that recollection involves the prefrontal cortex, the hippocampus and parahippocampus which encode and bind (i.e., associate one stimulus with another) relations between components of events [7],[8], [6]. Familiarity, on the other hand, involves the perirhinal cortex, which encodes representations of individual items [6]. Thus, the crucial distinction between recollection and familiarity depends on the presence or absence of contextual associations [9] and can be tested using paradigms that specifically probe memory for items and memory for associations (see [10–13]). Task analysis of these paradigms suggests that whereas items recognition may rely on both familiarity and recollection, associative recognition dominantly relies on recollection processes (see [12] for review). To date, there is a lack of studies testing the effect of NEA on neutral, non-autobiographical memory for items and for associations in healthy as well as in pathological populations.

The limited number of existing studies testing the impact of arousal on memory binding show mixed results. As an example, arousal (as induced by the application of a nociceptive stimulus after the presentation of neutral scenes) has been reported to enhance memory consolidation for neutral scenes [14]; in contrast, [15], suggested that as compared to positive and medium-neutral, negative arousal stimuli impaired memory for information that was the target of perceptual suppression, e.g. background information when there is a figure-ground distinction, but did not impair memory for other foreground information. While these studies tested memory for neutral stimuli learned under emotionally arousing context, emotional and arousing stimuli have also been reported to impact memory when the stimuli to be remembered are themselves arousing. For example, arousing stimuli (negative and positive, as compared to non-arousing stimuli) enhance the binding of an arousing picture's content to its location without interfering with picture-location binding for nearby pictures [16]. Examining the relative contribution of valence and arousal, [17] reported that both dimensions increased the vividness of remembered items, with greater effect for arousal than for valence only (this view is also supported by [18]). [19] showed that positive arousing pictures were more likely to be recalled as compared to non-arousing pictures by both young and adult healthy participants. In another study, emotionally-valenced words were reported to elicit enhanced free-recall compared with non-valenced words; however, recognition memory was not affected [20]. In the same study, associative memory was also enhanced for emotional words, suggesting that even memory for contextual information is benefited by emotional stimuli. In contrast, Nashiro and Mather [21] found that arousal improved within-items memory binding in younger adults but not in older adults, and additionally worsened both groups' between-items (associative) memory binding.

These studies focused on the impact arousal has on memory binding of emotionally-arousing stimuli and neutral stimuli/background location. While these studies are important to the understanding of how arousal impacts on memory, they were not designed to test intentional learning (that is, they established incidental learning memory paradigms) and they also involved additional parameters (such as the conjunction of an arousing picture and its background/location) which may also have contributed to the memory impairments observed under the arousal conditions. Most importantly, these studies were not designed to differentiate between the arousal component and the stimuli tested, and in addition, used the same stimuli modality for both the manipulation and the target stimuli (e.g. both the arousing stimuli and the target stimuli were pictures). Thus most studies are not able to point whether memory impairments occur due to a deficit in memory, or a specific deficit in emotional memory, or even due to attention deficits; nor they are able to point on the mechanism underlying these deficits (i.e. whether memory deficits occur due to impaired familiarity or recollection processes).

Recent studies conducted on populations with disorders associated with traumatic stress (see [10], [11], [13]) raised the hypothesis that associative memory impairments occur because of impaired associative binding process, presumably due to impaired recollection, i.e. when an encoded unit is retrieved with partial/without its contextual information [12]. This impairment in binding individual sensory features into stable objects or episodic memory may lead to fragmented recollection as expressed in incomplete/false memories [22], as seen in populations with disorders associated with traumatic stress. Among the main drawbacks of the majority of studies conducted with patients is their retrospective nature, often including participants with co-morbid disorders [23] who have been symptomatic for years with a wide variation in the time elapsed since the trauma and medication effects; all these factors may cause changes in cognition that are not strictly related to stress symptomatology [24]. These methodological issues make it difficult to draw firm conclusions regarding the effect of stress or its core symptoms (particularly arousal) on cognition and specifically, on associative memory [23], [25], thus require supporting evidences from analog studies conducted with healthy participants.

Focusing on the distinction between familiarity and recollection under arousing conditions in healthy participants may contribute to the understanding of memory impairments in populations with disorders associated with traumatic stress [22], [26], [27], and serve as a potential route to uncover the mechanisms underlying memory fragmentation in clinical populations [10], [11], [13]. Thus, in the current study we aimed to expand the understanding of the effect of acute NEA on recollection, by probing associative memory performance in healthy participants using an item-association memory paradigm, which allows distinguishing between items recognition (which is familiarity-based) and associative recognition (which is fundamentally based on recollection processes). In accordance with our previous results in patients, we hypothesize that under acute arousal conditions memory retrieval is based more on familiarity than on recollection processes, resultant in low accuracy and low associative recognition.

## Experiment I

### Methods

#### Participants

Participants in the current study were 30 psychology students from Achva Academic College that were rewarded for their participation with course credit, an acceptable procedure in a first-year introductory psychology academic course. Participants (4 and 3 males in the arousal and the non-arousal, i.e. arousal and control groups, respectively) were randomly assigned to one of two groups (arousal/control, n = 15 in each group). Mean age and education did not differ between groups (*M* = 23.84, *SD* = 1.62 and *M* = 23.60, *SD* = 1.59, *t*(28) = 0.23, ns; M = 12.13, SD = 0.35 and M = 12.26, SD = 0.45, *t*(28) = 0.89, ns, for age and education in the arousal and the control groups, respectively). All participants reported being in good health. Exclusion criteria included current sensory/motor disorders (participants with corrected vision were able to participate in the study) and past/current psychiatric or neurological disorders (as was confirmed by the participants in a self-report).

#### Ethics Statement

The study was approved by the local institutional review board of Achva Academic College. All participants gave their written informed consent for study participation.

### Stimuli and procedure

#### Memory task

To probe for items and associative memory we employed a modified procedure of our previously used item-association paradigm (see [10], [11], [13]). All participants were given standardized instructions prior to the beginning of the memory paradigm. Overall, participants performed one block of training (that included a learning list, followed by items and association recognition tests), and four repetitions of blocks with a similar design.

**Fig 1:**
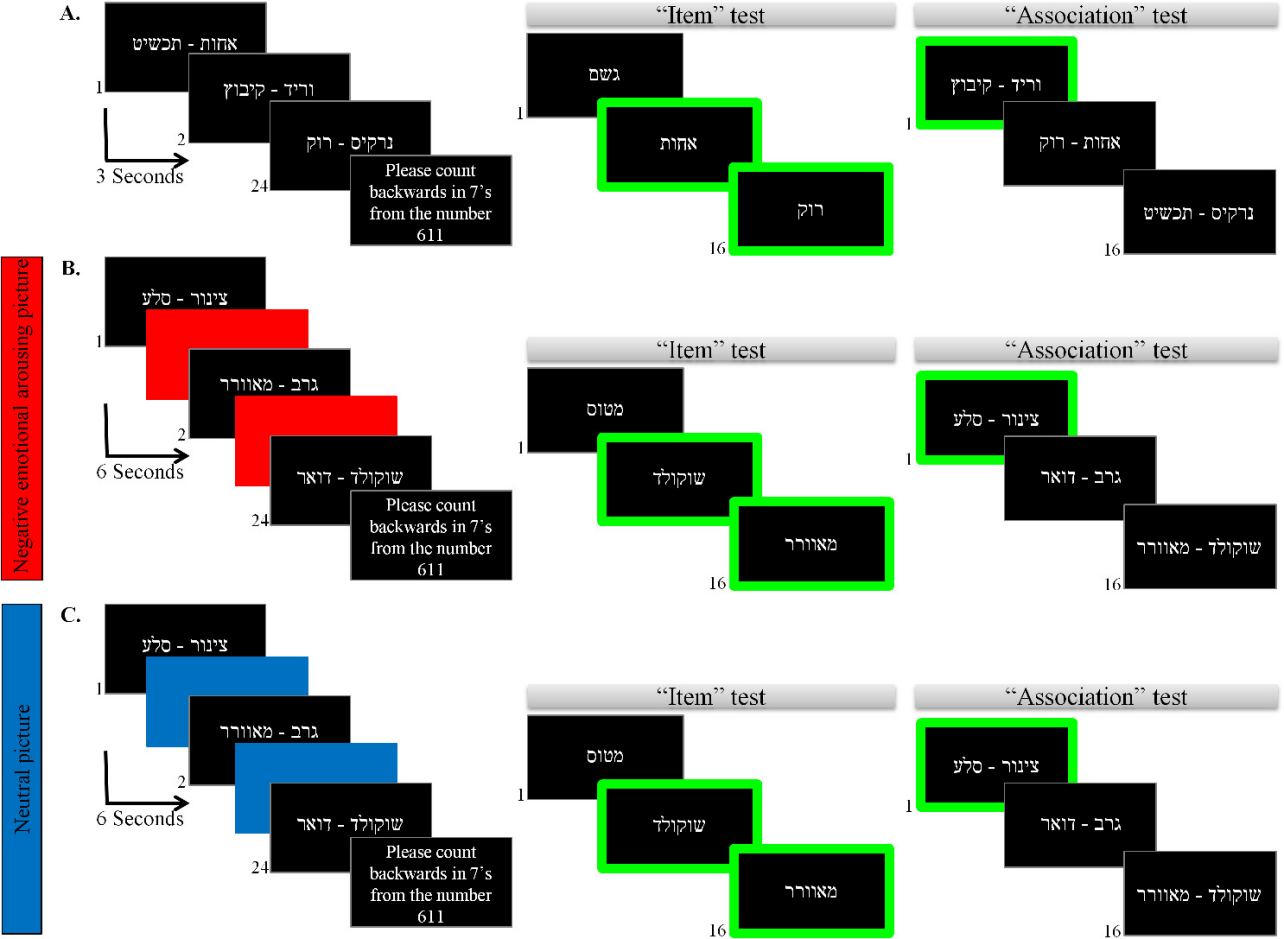
Experimental paradigm. Participants were presented with a study list of 24 unrelated (visually or semantically) emotionally neutral pairs of words, one at a time. The first two lists (A) were used as baseline while the two remaining lists were learned under negative emotional arousal (B) / neutral (C) condition. Participants were instructed to respond to each stimulus on the keyboard with a designated “yes” key (= 1) for targets and a “no” key (= 0) for distracters.

In each learning phase, participants were asked to study a list of 24 pairs of unrelated emotionally-neutral words, presented on a 15'' computer monitor (different stimuli were used in each learning phase and list). Stimuli were randomized across participants and were compiled from high-frequency two- or three-syllable concrete nouns [28], and were not related semantically or rhythmically (i.e. " hair—chocolate"). In the non-arousal (i.e. control) group, stimuli pairs were presented at a rate of 3 seconds per pair (inter-stimulus interval = 1 second, thus the overall interval between the onset of stimuli pairs was 4 seconds). In the arousal group, stimuli pairs were presented at a rate of 3 seconds per pair (inter-stimulus interval = 1 second, IAPS picture presentation = 2 seconds prior to each pair, thus the overall interval between the onset of stimuli pairs was 6 seconds). Learning was intentional: participants were instructed to learn both the individual stimuli and the pairs. The learning phase was followed by a 30-second distraction task (counting backward in sevens from a randomly selected number) to prevent rehearsal between the learning and memory-task. Participants performed the items test, followed by the associative test. The first two lists were used as baseline for the evaluation of the participant's items and associative memory performance, while the other two lists were learned under neutral (for controls) or negative (for the NEA group) IAPS manipulation. The lists were randomly assigned as baseline or experimental lists for each participant. The experimental paradigm is shown in Fig 1.

Items recognition test: In this test, participants viewed 16 words on the computer monitor, one at a time. From these, eight were targets (i.e. appeared in the learning phase) and eight were distracters (i.e., new stimuli that had not appeared in the learning phase, but shared similar characteristics as the target stimuli), mixed randomly. Participants were informed that the list included targets and distracters, and were instructed to respond to each stimulus with a designated “yes” key for targets and a “no” response key for distracters.

Associative recognition test: In this test, participants viewed 16 word-pairs on the computer monitor, one at a time. Eight of these were intact pairs from the learning phase (i.e. the same pairs that appeared in the learning phase) and the other eight pairs were rearranged pairs (i.e. contained the same items from the learning list that were now recombined and presented as novel pairs), As in the items recognition test, participants were instructed to respond by pressing the “yes” key for targets, and the "no" key for the distracters (rearranged pairs). Stimuli that were used in the items test were not used in the associative test and vice-versa.

Scoring: To assess differences in performance between the items and associative recognition tests, we computed a measure for the percentage of hits (responding "yes" to a target) minus the percentage of false-alarms (responding "yes" to a distracter) for each participant and experiment. This measure of memory accuracy equated the scales for the items and the associative recognition tests, with chance level performance at 0.00 and perfect performance at 1.00.

Operationalization of items and associative recognition as in the current study enables distinguishing between familiarity-based and recollection-based processes. Since episodic memory is based on familiarity and recollection [6], operationalizing items and associative recognition distracters in the above manner allows drawing a line between items-recognition performance (which is familiarity-based) and associative performance (which is fundamentally based on recollection processes as stated in a review by [12]). That is, in order to perform well in the associative test it is not enough to be familiar with two items, rather one must recollect enough details to determine whether those items were studied together or not.

#### Negative emotional arousal versus neutral pictures manipulation

Stimuli used to manipulate NEA versus neutral condition were pictures from the International Affective Picture System (IAPS, [29]). Pictures for each condition (highly negative arousing pictures and low positive arousing pictures for the arousal versus the neutral condition, respectively) were chosen according to their valence and arousal ratings. The mean valence and arousal ratings of the pictures used in the NEA group were 2.04 (SD = 0.47) and 6.34 (SD = 0.82), respectively, and 6.83 (SD = 0.63) and 3.52 (SD = 0.61) in the control group.

After the two baseline memory measures, the NEA/neutral pictures induction phase has began. Participants in the NEA group were requested to read a sham article describing a major car accident with a high number of casualties (participants were told that the article was taken from the weekly newspaper) while participants in the control group received a sham article describing a weekend picnic. After the participants finished reading the article, they were requested to complete another two blocks of tests, only in the current blocks we added a picture before each word-pair (presented for 2 seconds) in the learning phase. Participants were told that these pictures were taken from the article they read before the memory tests.

#### Manipulation check

To asses the anxiety levels we used the Spielberger's State Anxiety Inventory (SAI); a self-report questionnaire that measures state anxiety. The SAI questionnaire contains 20 items and each item is rated on a 4-point Likert scale (range from '1' = not at all to '4' = very much). The total scores of this measure are obtained by summing the values assigned to each item, and range from a minimum of 20 to a maximum of 80, with higher scores indicating more severe anxiety symptoms [30]. Participants filled the questionnaire a few minutes before the baseline phase and after the end of the experiment. Participants were given standardized instructions prior to completing the STAI questionnaire.

### Results

#### Manipulation Check

Internal consistency for the SAI questionnaire was calculated using Cronbach alpha (= .855). Since no main effect or interactions with gender were found, all reported results are collapsed over gender. No significant difference in the Spielberger State Anxiety Inventory (SAI; [30]) was found between groups before the manipulation (*M_NEA_* = 28.26 *SD* = 6.57, *M_control_* = 29.13 *SD* = 6.32, *t*(28) = 0.36, n.s.). To assess the effectiveness of the NEA/neutral manipulation we tested the differences in the SAI before and after the manipulation. The results of the two-way mixed-design analysis of variance (ANOVA) [*Group* (NEA, Control) X *Time* (Pre/Post experimental phase)] with repeated-measures on the second factor (*Time*) indicated a close to significant main effect for *Group* (F(1, 28) = 4.19, p = .05, ηp2 = .13); a significant main effect for *Time* (F(1, 28) = 9.44, p = .004, ηp2 = .25) and more importantly, the interaction of these two variables was significant (F(1, 28) = 15.47, p = .000, ηp2 = .36). Planned comparisons analysis reflected that while no differences between the pre-to-post experimental phase were evident in the control group (*M_before_* = 29.13 *SD* = 6.32, *M_after_* = 27.66 *SD* = 5.97, *t*(14) = 1.50, n.s.) a significant elevation in the SAI levels was observed in the NEA group (*M_before_* = 28.26 *SD* = 6.57, *M_after_* = 40.20 *SD* = 14.55, *t*(14) = 3.65, p = .000, ηp2 = .41) (see also Table 1).

**Table 1:** Anxiety levels (SAI) and Memory Performance in the Pre and Post Manipulation Phases for the negative emotional arousal and Control Groups.

		Pre-Manipulation		Post- Manipulation		Pre-Neutral		Post-Neutral	
		M	SD	M	SD	M	SD	M	SD
SAI ^a^		28.26	*6*.*57*	40.20	*14*.*55*	*29*.*13*	*6*.*32*	*27*.*66*	*5*.*97*
Mean Items Recognition	Hits	0.73	*0*.*15*	0.72	*0*.*17*	*0*.*79*	*0*.*16*	*0*.*80*	*0*.*14*
	FA	0.11	*0*.*09*	0.19	*0*.*11*	*0*.*13*	*0*.*10*	*0*.*15*	*0*.*11*
Mean Associative Recognition	Hits	0.79	*0*.*14*	0.68	*0*.*14*	*0*.*80*	*0*.*18*	*0*.*78*	*0*.*16*
	FA	0.21	*0*.*18*	0.35	*0*.*06*	*0*.*21*	*0*.*19*	*0*.*36*	*0*.*14*

^a^ SAI = Spielberger State Anxiety Inventory

#### Memory performance

**Fig 2:**
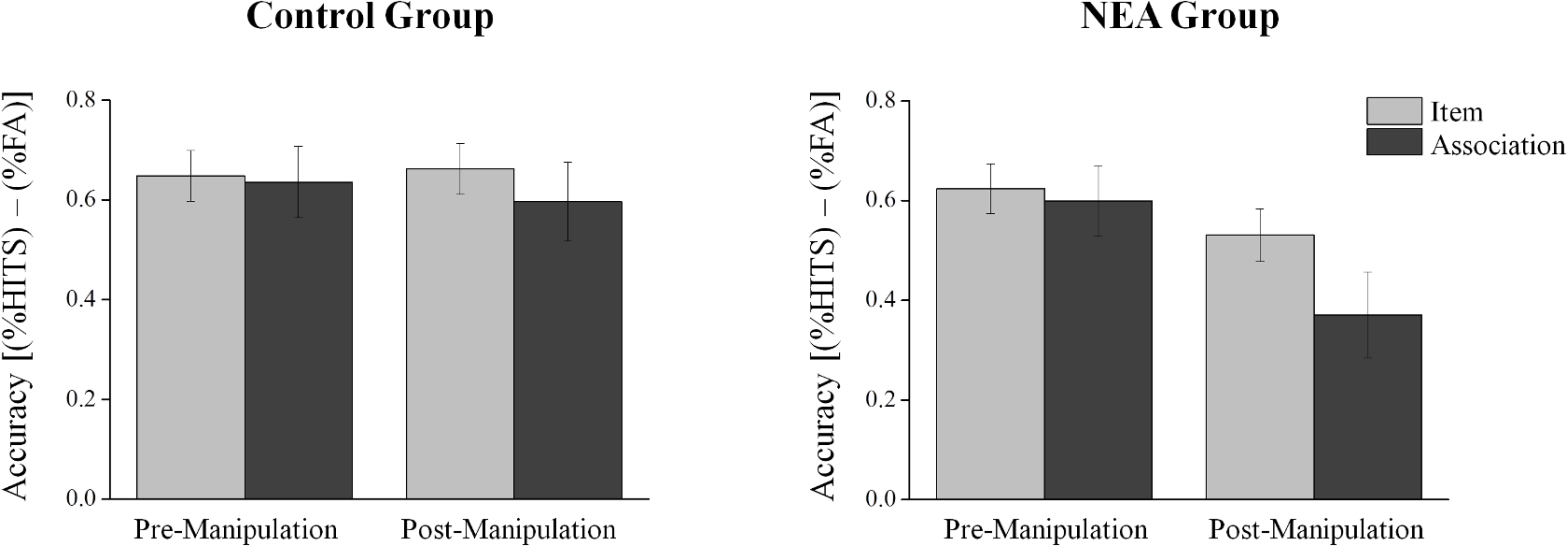
Behavioral results. Memory performance on items and the associative recognition tests in the pre-and post-negative emotional arousal (NEA) manipulation phases in each group. The measure used is proportion hits minus proportion false alarms (FA). Error bars represent the standard error of the mean.

The mean proportions of hits and false alarms for each task (items and associative recognition) and experimental phase (pre-post manipulation) are shown in Table 1. Since a preliminary analysis yielded no interaction effect of gender with any of the independent variables, all results reported below are collapsed over gender. To specifically address the hypothesis tested in this experiment we employed a three-way mixed-design ANOVA [*Group* (NEA, Control) *X Time* (Pre/Post experimental phase) *X Task* (Items, Association)] with repeated measures on the second and third factors (*Time* and *Task*) and the dependent variable as *Memory accuracy*. The ANOVA results are presented in Fig 2. As expected, a significant main effect for *Task* was found (*F*(1, 28) = 5.08, *p* = .032, *η_p_^2^* = .15) with the items recognition (*M* = 0.62, *SD* = 0.18) surpassing the associative recognition performance (*M* = 0.55, *SD* = 0.28) regardless of *Time* and *Group*. A second significant main effect was found for *Time* (*F*(1, 28) = 12.94, *p* = .001, *η_p_^2^* = .32), with the memory performance at the pre-manipulation phase (*M* = 0.63, *SD* = 0.22) was higher as compared to the post- manipulation phase (*M* = 0.54, *SD* = 0.25), regardless of *Task* type and *Group*. The main effect for *Group* was not significant (*F*(1, 28) = 1.69, *p* = .20, *η_p_^2^* = .06). The two-way interactions (*Time X group*, *Time* X *task*) were significant (*F*(1, 28) = 9.41, *p* = .004, *η_p_^2^* = .25; and *F*(1, 28) = 5.12, *p* = .031, *η_p_^2^* = .15, respectively). Although the 3-way (*Group X Time X Task*) interaction was not significant (*F*(1, 28) = .96, *p* = .337, *η_p_^2^* = .03), we performed our planned comparisons to test the simple-interaction between *Task* and *Time* separately for each group (Significant ANOVA results are not a pre-condition for performing focused contrasts (see Rosnow & Rosenthal, [31])). We report a significant simple-interaction in the NEA group (*F*(1, 28) = 5.25, *p* = .029, *η_p_^2^* = .16), while the interaction in the control group was not significant (*F* < 1, n.s.). Further planned comparisons on this interaction effect showed no significant associative deficit (i.e. a decrease between items and associative recognition) before the NEA manipulation (F < 1, n.s.) while a significant associative deficit was observed in the NEA group after the manipulation (*F*(1, 28) = 8.77, *p* = .006, *η_p_^2^* = .24) (see Fig 2).

To summarize, while no differences in anxiety levels were found between groups in the pre-experimental phase, anxiety levels at the post-manipulation phase in the NEA group were higher than in the control group. Testing for memory accuracy indicated that items were better remembered than associations and that memory performance in the pre-manipulation phase was better as compared with the post-manipulation phase in both groups. Most importantly, the NEA group displayed an associative deficit in the post-manipulation phase that was not evident in the control group (the 3-way interaction was not significant).

## Experiment II

The results of the first experiment confirmed our hypothesis by indicating an associative memory deficit in healthy young adults under NEA condition. In order to increase power, we conducted an additional experiment in which we included more participants, adapted the learning phase and applied a more engaging manipulation. This procedure was designed to allow further generalization of the results and in order to strengthen the convergent findings from the first experiment (i.e. confirm that the results from the first experiment are not a consequence of a specific NEA operationalization). Specifically, the current experiment serves as a constructive replication of the first experiment with the following modifications: first, while in the first experiment NEA was manipulated by flashing the IAPS pictures in the learning phase after each pair of stimuli (within list), in the current experiment we manipulated arousal between lists and in addition, asked the participants to write a story regarding the IAPS picture instead of reading an article. These changes were made in order to increase participant's engagement in the task and thus to test a more pronounced effect of NEA on memory. In the current experiment the learning phase included 36 word-pairs and participants were tested on 12 stimuli (items/associative pairs, for the items and associative tasks, respectively). Furthermore, since studies have demonstrated that stress, as-well-as its core symptoms- e.g. arousal, can facilitate the hyperactivity of the sympathetic division of the autonomic nervous system, including heart rate responses (HRR) [32–35], in the current experiment we used HR levels as an objective physiological measure of arousal.

### Methods

#### Participants

Participants in the second study were 50 psychology students from Achva Academic College which were rewarded for their participation with course credit. Participants were assigned randomly to one of two groups, NEA and control (21 and 17 women in each group, respectively). Three participants were excluded from the analysis due to abnormal physiological measurements (see results section for more details), thus the following analysis was performed on 47 participants (n = 24, n = 23 for the NEA and control groups, respectively). Mean age and education did not differ between groups (*M* = 25.66, *SD* = 5.2 and *M* = 24.7, *SD* = 4.12, *t*(45) = 0.67, ns; M = 12.29, SD = 0.69 and M = 12. 65, SD = 1.49, *t*(45) = -1.06, ns, for age and education in the NEA and control groups, respectively). All participants reported being in good health. Exclusion criteria included current sensory/motor disorders (participants with corrected vision were able to participate in the study) and past/current psychiatric or neurological disorders (as was confirmed by the participants in a self-report).

In addition, Participants who took part in the first experiment were not allowed to participate in the current experiment.

#### Ethics Statement

The study was approved by the local institutional review board. All participants gave their written informed consent for study participation.

### Stimuli and procedure

#### Memory task

To probe for item and associative memory we employed a similar procedure as in the first experiment with the following changes: in the learning phase, participants were asked to study a list of 36 pairs of unrelated emotionally-neutral words, presented on a 15'' computer monitor, at a rate of 3 seconds per pair (inter-stimulus interval = 1 second) and were randomized across participants. As in the first experiment, participants performed the items task, followed by the associative task. Learning was intentional: participants were instructed to learn both the individual stimuli and the pairs. The learning phase was followed by a 30-second distraction task (counting backward in sevens from a randomly selected number) to prevent rehearsal between the learning and memory-task. Participants performed the items test, followed by the associative-test. The first two lists were used as baseline for the evaluation of the participant's items and associative memory performance, while the other two lists were learned after the NEA/neutral manipulation. The lists were randomly assigned as baseline or to the NEA condition for each participant.

Items recognition test: In this test, participants viewed 12 words, on the computer monitor, one at a time. Of these, six were targets and six were distracters (i.e., had not appeared in the learning phase), mixed randomly. Participants were informed that the list included targets and distracters, and were instructed to respond to each stimulus with a designated “yes” key for targets and a “no” response key for distracters.

Associative recognition test: In this test, participants viewed 12 word-pairs on the computer monitor. Six of these were intact pairs from the learning phase and the other six pairs were rearranged pairs; that is, all the items had appeared in the learning phase but half of the items were now recombined into novel (distracter) pairs. As in the items recognition test, participants were instructed to respond by pressing the “yes” key for targets, and the "no" key for distracters (rearranged pairs).

#### Negative emotional arousal versus neutral pictures manipulation

After the two baseline memory blocks, the induced NEA phase has began. Participants were requested to attend a highly negative arousing picture (taken from the IAPS archive) for 30 seconds. Participants were requested to remember as many details as possible regarding the picture and not to block any feelings raised by it. At the end of the 30 seconds participants were requested to write a story about the picture that will include information regarding the situation before and after the picture as well as what led to this picture for 2.5 minutes. Control subjects underwent the same procedure with a neutral (i.e. non-arousing) picture.

#### Heart Rate measure

HR was measured using the “Freeze framer” system (version 2.0) that measured the HR levels by connecting a fingertip pulse-sensor to the subject’s finger on the non-dominant hand. To reduce noise as a result of hand movements, subjects were requested to place the hand connected to the sensor on their knee and avoid unnecessary movements. HR measurement lasted two minutes and was conducted twice; at baseline (i.e. the first two blocks of the memory task) and while the manipulation phase (i.e. while subjects were writing their story about the picture).

### Results

One participant from the NEA group and two participants from the control group were excluded from the analysis due to high HR bit (above 100 p/m) at the baseline measure, thus, the following results are reported for 47 participants (24 and 23 for the NEA and control groups, respectively). Additionally, since no main effect or interactions with gender were found, all reported results below are collapsed over gender.

#### Heart Rate measure

HR measures before and after IAPS presentation in each group are presented in Table 2. No significant difference in HR was found between groups before the manipulation (*M_NEA_* = 81.89 *SD* = 8.71, *M_control_* = 82.34 *SD* = 6.98, *t*(45) = 0.19, n.s.). A two-way mixed-design ANOVA [*Group* (NEA, Control) X *Time* (Pre/Post experimental phase)] with repeated-measures on the second factor (*Time*) revealed that the *Group X Time* interaction approached significance (*F*(1, 45) = 3.13, *p* = .08, *η_p_^2^* = .06). In addition, the results indicated a significant main effect of *Time* (*F*(1, 45) = 6.50, *p* = .01, *η_p_^2^* = .12), but a non-significant main effect of *Group* (*F* < 1). Comparing HR before and after IAPS presentation in the NEA group yielded a significance increase, demonstrating elevated HR levels in this group (*M_before_* = 81.89 *SD* = 8.71, *M_after_* = 87.08 *SD* = 11.30, *t*(23) = 2.53, p = .018, ηp2 = .48) with no significant HR elevations in the control group (*M_before_* = 82.34 *SD* = 6.98, *M_after_* = 83.28 *SD* = 6.56, *t*(22) = 0.77, n.s.).

**Table 2:** Heart-Rate and Memory Performance in the Pre and Post Manipulation Phases for the negative emotional arousal and Control Groups.

		Control group				Negative Emotional Arousal Group			
		Pre Manipulation		Post Manipulation		Pre Manipulation		Post Manipulation	
		M	SD	M	SD	M	SD	M	SD
Heart Rate		82.34	*6*.*98*	83.28	*6*.*56*	81.89	*8*.*71*	87.08	*12*.*30*
Mean Item Recognition	Hits	0.71	*0*.*18*	0.64	*0*.*19*	0.70	*0*.*15*	0.68	*0*.*16*
	FA	0.23	*0*.*18*	0.20	*0*.*18*	0.19	*0*.*13*	0.19	*0*.*20*
Mean Associative Recognition	Hits	0.67	*0*.*19*	0.69	*0*.*19*	0.74	*0*.*15*	0.62	*0*.*20*
	FA	0.26	*0*.*19*	0.23	*0*.*17*	0.18	*0*.*17*	0.16	*0*.*14*

### Memory performance

**Fig 3:**
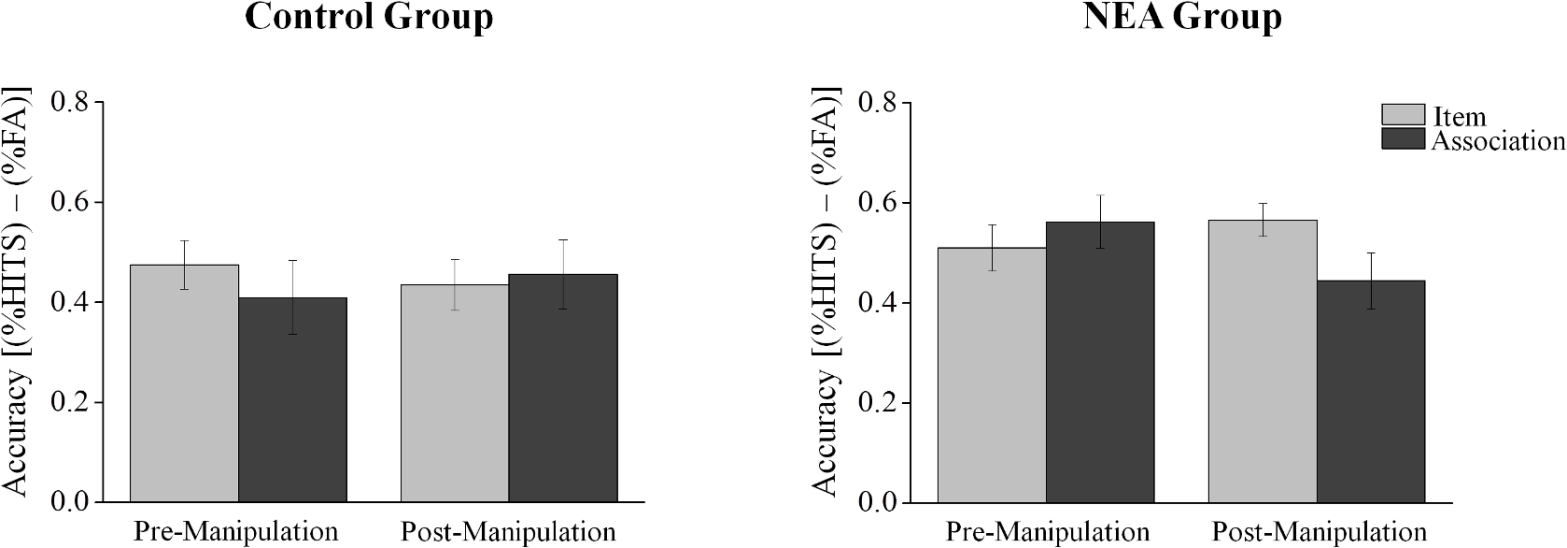
Behavioral results. Memory performance on items and the associative recognition tests in the pre-and post-negative emotional arousal (NEA) manipulation for each group. The measure used is proportion hits minus proportion false alarms, and the error bars represent the standard error of the mean.

The mean proportions of hits and false alarms for each memory task (items and associative recognition) and time (pre/post experimental phase, i.e., the IAPS presentation) are shown in Table 2. To specifically address the hypothesis tested in this experiment we employed a three-way mixed-design ANOVA [*Group* (NEA, Control) *X Time* (Pre/Post experimental phase) *X Task* (Items, Association)] with repeated measures on the second and third factors (*Time* and *Task*) and the dependent variable as *Memory accuracy*. The results (see Fig 3) indicated a significant three-way (*Group X Time X Task*) interaction (*F*(1, 45) = 10.66, p = .002, *η_p_^2^* = .19). While planned comparisons yielded no significant simple interaction between *Time* and *Task* in the control group (*F*(1, 45) = 2.31, p = .13 ns), this interaction in the NEA group was found to be significant (*F*(1, 45) = 9.70, p = .003, *η_p_^2^* = .18). Further analysis on this interaction effect yielded no difference between items and associative recognition performance before the NEA manipulation (*F* < 1, n.s.), but a significant decrease in associative recognition as compared to items recognition was observed after the NEA manipulation (*F*(1, 45) = 4.33, p < .043, η_p_^2^ = .09 see Table 2 and Fig 3).

To summarize, while no differences in HR levels were found between groups in the pre-experimental phase, a significant increase in HR levels was evident in the NEA group following the manipulation. Testing for memory accuracy in the NEA group indicated that while no difference was found between items and associative recognition performance before the NEA manipulation, a significant decrease in associative recognition was observed after the NEA manipulation.

## Discussion

The unique design of the current study adds another perspective regarding the effect of arousal on memory, synthesizing often disparate findings from individual studies by testing the effect of NEA on associative, emotionally-neutral, non-autobiographical information. The results of the two experiments show that NEA, as induced within (experiment 1) or between (experiment 2) study lists, impaired associative recognition. The results of the second experiment demonstrate elevated HR levels in the NEA group following the manipulation, confirming that NEA was indeed perceived by participants. These results provide an additional objective (physiological) measure, in addition to self-report anxiety levels, supporting the validity of the manipulation. Although in the current study we employed intentional learning of neutral target stimuli and manipulated arousal differently from previous studies ([20], [17], [15], [16], [19]) our findings are in line with the results of Nashiro and Mather [21] who reported that arousal worsened between-items (associative) memory. The current study clearly shows that while items recognition stayed preserved, associative recognition was impaired following the NEA manipulation.

Studying the relationship between arousal and between-items (associative) memory performance for neutral stimuli has recently emerged as a potential contributor to the understanding of cognitive alterations seen in disorders associated with traumatic stress, and specifically posttraumatic-stress disorder (PTSD). PTSD is mental disorder that may develop after a traumatic life event and is characterized by symptoms such as re-experiencing the traumatic event, avoidance of situations associated with it, negative mood and cognitions and hyper-arousal [36]. The majority of neuroimaging studies in PTSD report brain alterations in structures involved in binding components of events (e.g. the prefrontal cortex, the hippocampus and parahippocampus; see meta-analysis by Karl et al., [37]), and indeed previous studies in these populations have shown increased bias towards false-memories [38], [39], [40], [10], [11], [13], and more specifically higher rates of false recognition in associative memory tasks (see [10], [11], [13]). Since increased susceptibility to conditioning was also reported in PTSD [41] and it is known that one common tendency of PTSD patients is overgeneralization from traumatic cues to unrelated neutral ones [42], resultant in binding together negative and neutral stimuli, and erroneous associations between neutral stimuli it is therefore hypothesized that memory fragmentation in populations with disorders associated with traumatic stress may be related to an altered binding process. This alteration can in turn lead to impaired recollection, resulting in a tendency to mistakenly associate unrelated stimuli, as expressed in abnormal associative memory. This view of impaired recollection under acute arousal conditions is consistent with the "temporal dynamics” model suggested by Diamond et al. [43], which holds that the onset of strong emotionality (i.e. arousal or stress), leads the hippocampus to rapidly shift from a “configural/cognitive map” mode to a “flashbulb memory” mode, which underlies the long-lasting, but fragmented, nature of traumatic memories. The findings of the current study are in line with this model, showing impaired binding process under the NEA condition in healthy participants.

Several limitations of the current study must be acknowledged. Firstly, whether or not the current study results can be generalized to additional populations (e.g. males, older populations etc.) remains an open question, as our sample consisted mainly of young women (73% and 80% in the first experiment, 84% and 68% in the second experiment, for the NEA and control groups respectively), which have been reported to show a stronger negative effect of psychosocial stress on memory as compared to men [44], [45]. Secondly, the current design precludes reaching definitive conclusions regarding whether the effect of NEA on memory is due to impaired encoding, or retrieval, or both. Further replications of the current study are required in order to answer these questions. In addition, since anxiety scores were collected before baseline and after the manipulation, it is possible that the post-assessment reflects anxiety levels resulting from both the baseline and the experimental phases. Although the two anxiety scores did not differ in the control group, further studies should consider adding an additional assessment before the experimental phase.

An important distinction must be made between trauma processing, as seen in populations with disorders associated with traumatic stress, and the processing of NEA-related stimuli, as seen in healthy participants in the current study. The processing of the negative arousing stimuli was conducted under "sterile" lab-conditions, and may be fundamentally different in nature from the processing of a traumatic event in everyday life as experienced by PTSD patients. Although the current analogous study results must be interpreted with caution in the context of pathological populations, overall, we have demonstrated that under laboratory conditions NEA impaired memory performance, as expressed in altered recollection. This deficiency was specific to associative memory–an aspect of memory which has previously been shown to be impaired in population with disorders associated with traumatic stress. In light of our previous findings in patients and the current findings in healthy participants, we suggest that although not all individuals exposed to a traumatic event will develop PTSD, exposed individuals may present associative memory deficits, even for neutral, non-trauma related stimuli. This hypothesis is consistent with the "temporal dynamics" model of Diamond et al. [43] and with therapeutic approaches to PTSD that target the integration of fragmented memories into a cohesive episode and narrative.

## Supporting Information

S1 File Complete Data Set used for data analysis in experiment 1. (XLS)

S2 File Complete Data Set used for data analysis in experiment 2. (XLS)
